# Accuracy of Retinal Pigment Epithelium (RPE) undulations (Oscillatory sign) as an optical coherence tomography biomarker of acute RPE tears

**DOI:** 10.1186/s40942-026-00874-7

**Published:** 2026-06-20

**Authors:** Manoj P. Shettigar, Jay Chhablani, Mudit Tyagi, Ritesh Narula, Raja Narayanan

**Affiliations:** 1 Wen Giving Foundation Center for Vitreo-Retinal Diseases, Anant Bajaj Retina Institute, LV Prasad Eye Institute, Kadapa, India; 2 University of Pittsburgh, UPMC Eye Center, Choroidal Analysis and Research (CAR) Lab, Pittsburgh, USA; 3 Smt. Kanuri Santhamma Center for Vitreo Retinal Diseases, Kallam Anji Reddy Campus, Anant Bajaj Retina Institute, L V Prasad Eye Institute, Hyderabad, India; 4 Flaum Eye Institute, University of Rochester Medical Center, Rochester, NY 14642 USA

**Keywords:** Neovascular age-related macular degeneration, Retinal pigment epithelium tears, RPE undulations, Oscillatory sign

## Abstract

**Purpose:**

To evaluate the sensitivity and specificity of Retinal Pigment Epithelium (RPE) undulations (Oscillatory sign) on Optical Coherence Tomography (OCT) and assess its potential role as a predictive OCT biomarker for RPE tears in eyes with neovascular age-related macular degeneration (nAMD).

**Methods:**

Retrospective analysis of the patients who had RPE tears following nAMD, which were documented by multimodal imaging. All cases were evaluated for RPE undulations on OCT, and the sensitivity and specificity of the Oscillatory sign in eyes with documented RPE tears were assessed.

**Results:**

Seventy-nine eyes out of 936 eyes with nAMD, which were analyzed, were found to have RPE tears, as documented by colour fundus photographs (CFP) and fundus autofluorescence (FAF). OCT images were analyzed for RPE undulations, a sign of oscillatory activity. Thirty-eight eyes of 79 (48.1%) eyes were found to have the oscillatory sign. Oscillatory sign showed a high specificity of 98.95% and sensitivity of 48.10% for an underlying RPE tear. The negative predictive value of the Oscillatory sign was 95.39%, and the positive predictive value of 80.85%.

**Conclusion:**

The Oscillatory sign of RPE undulations is not sensitive, but it is a highly specific biomarker and can help facilitate the diagnosis of occult RPE tears.

## Introduction

Retinal pigment epithelium tears (RPE) are a devastating complication often associated with neovascular age-related macular degeneration (nAMD). RPE tears can lead to progressive loss of the retinal RPE, photoreceptors, and the underlying choroid, with a profound impact on visual function. RPE tears are relatively frequent, often associated with vascularized pigment epithelial detachments (PED), with reported incidence rates of 10% to 15% in eyes with neovascular age-related macular degeneration (nAMD) [1–3]. Visual acuity is often impaired in these patients, particularly when larger tears are present, and the foveal center is involved [2, 3]. It can occur as part of natural history or after using intravitreal anti-vascular endothelial growth factor (VEGF) therapy [4]. 

Retracted RPE resulting from an RPE tear results in increased autofluorescence due to increased lipofuscin from scrolled RPE. This enhancement makes fundus autofluorescence (FAF) a particularly sensitive imaging modality for diagnosing large RPE tears [5, 6]. The distinct contrast between the hypoautofluorescent areas and the surrounding intact retina significantly aids in accurately identifying lesion boundaries in FAF imaging. While autofluorescence is valuable, it may not detect small RPE tears or those that are impending. OCT is a more commonly used imaging method among ophthalmologists. OCT of eyes with RPE tears has been described earlier, showing subtle RPE discontinuities that may go unnoticed in FAF [7–9]. Additionally, OCT can illustrate the retracted edges of RPE tears, which are depicted as hyperreflective structures. In the literature, “Wavy RPE indentations” have been used as a predictor of RPE tears following anti-VEGF injections [10, 11]. The aim of this study is to describe the novel Oscillatory sign on OCT and to analyze its accuracy in diagnosing RPE tear in nAMD.

## Methods

### Study design and ethics

This was a retrospective, consecutive, non-comparative descriptive study conducted at a tertiary eye center in India between March 2017 and March 2025. The study was approved by the Institutional Review Board (LEC-BHR-R-029) and adhered to the tenets of the Declaration of Helsinki. All participants provided informed consent for the collection of electronic data for research purposes. The study was not masked, and a single observer (M.P.S.) performed the analysis.

### Patient selection and data retrieval

We reviewed the electronic medical records of patients diagnosed with nAMD. Inclusion criteria were: [1] The cohort includes all patients with neovascular AMD [2] the presence of an RPE tear at presentation or following treatment initiation, and [3] documented colour fundus photographs (CFP) with or without FAF. Data on eyes with RPE tears at presentation or after initiation of treatment were included in the study.

Exclusion criteria included RPE tears secondary to other causes, such as Central serous chorioretinopathy, OCT scans with poor signal strength or artifacts, and cases where the diagnosis of RPE tear was uncertain.

### Clinical and imaging protocol

All participants underwent a comprehensive ocular examination. Snellen visual acuity measurements were recorded and converted to logMAR notation for statistical analysis.

Multimodal imaging was performed using the following devices:

- OCT: Zeiss Cirrus HD-OCT (Carl Zeiss Meditec, Dublin, CA, USA); DRI OCT Triton (Topcon, Tokyo, Japan); or Spectralis OCT (HRA-2, Heidelberg Engineering, Heidelberg, Germany).
- FAF: Heidelberg Retina Angiograph (HRA-2, Heidelberg Engineering).
- CFP: Clarus 700 true colour images (Carl Zeiss Meditec, Dublin, CA, USA).

### Image analysis and sign definition

**Fig. 1:**
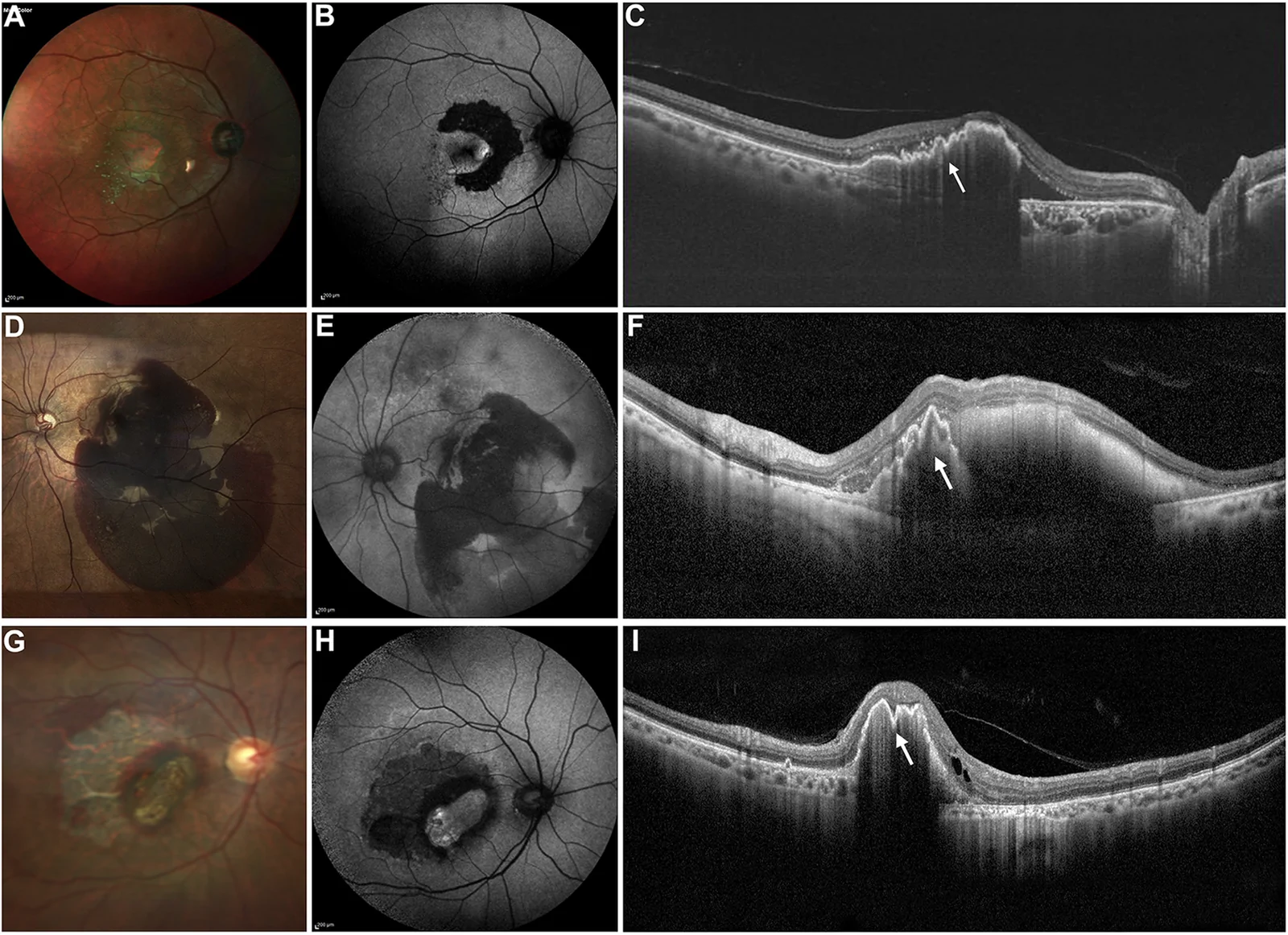
Multimodal imaging of RPE tear. Case (1) **A**. Multicolour fundus photograph of an 82-year-old male showing the presence of a well-delineated RPE tear. **B**) Fundus autofluorescence (FAF) showing a crescent-shaped hypoautofluorescent area due to RPE tear. **C**. OCT shows an area of absence of RPE reflectivity with an adjoining area of RPE undulations. (Arrowhead) Case (2) **D**) Fundus photograph of a 62-year-old female showing the presence of extensive subretinal hemorrhage. **E**) FAF photos showing blocked autofluorescence areas due to subretinal haemorrhage. **F**) Spectral-domain OCT shows a large area devoid of RPE with an adjoining area of hyperreflectivity in the area of RPE undulations. (Arrowhead) Case 3: **G**) A Fundus photograph of the right eye of a 65-year-old male with RPE tear and subretinal hemorrhage. **H**) FAF photo showing hypoautofluorescent areas due to a denuded area of RPE tear. **I**) Spectral-domain OCT shows pigment epithelial detachments (PED) with focal disruption of RPE and an area devoid of RPE reflectivity, with an adjoining area of high reflectivity and an adjoining area of RPE undulations. (Arrowhead)

**Fig. 2:**
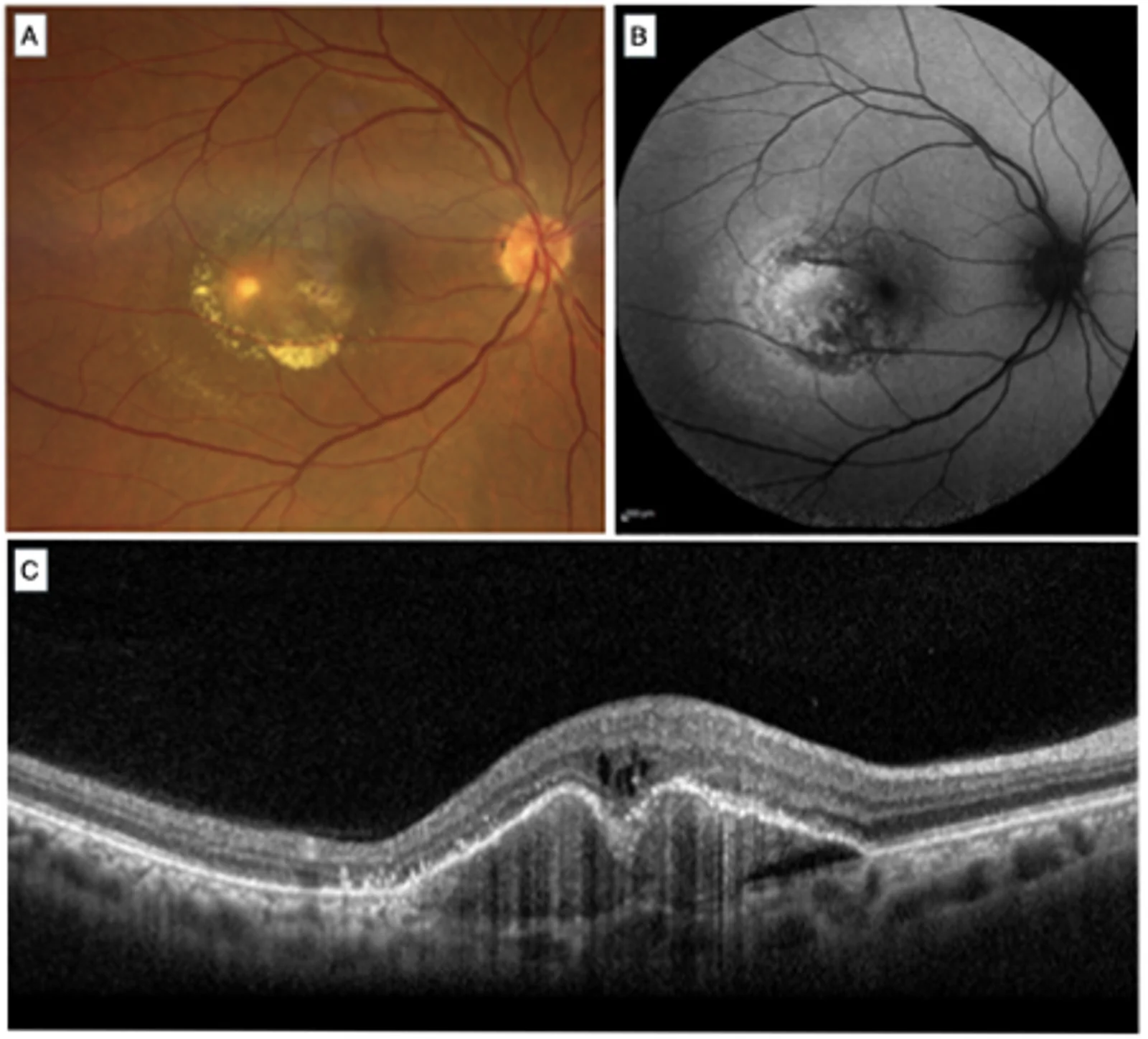
Multimodal imaging of RPE tear. Case 4. (**A**) Colour fundus photograph of a 60-year-old male showing the exudates temporally and inferior to the fovea. (**B**) Hyperautofluorescence temporal to the fovea and a linear hypoautofluorescent area superior to the fovea due to RPE tear. (**C**) Spectral-domain OCT showing a notch in PED with intraretinal fluid with discontinuous RPE suggestive of an RPE tear with no RPE undulations, consistent with the no-oscillatory sign

CFP and FAF served as the reference standard for analysis. On CFP, an RPE tear was diagnosed by a well-defined hyperpigmented line at the rolled RPE site and a depigmented area of exposed choroid (Fig. 1A). On FAF, **t**he margins were determined precisely by the great contrast of these hypoautofluorescent regions compared to the uninvolved retina. (Fig. 1B). Horizontal and vertical raster scans of OCT scans were specifically analyzed for the Oscillatory sign over an existing PED. The oscillatory sign is characterized by RPE scrolling with undulations, hyperreflectivity, and backshadowing at the rolled edges of the RPE. This is accompanied by increased hypertransmission in the area of the RPE defect (Fig. 1C). Scans passing perpendicular to the angle of the RPE tears were explicitly evaluated. A RPE tear with “no-oscillatory sign” was defined as an area of denuded RPE with adjacent rolling but lacking RPE undulations. (Fig. 2). OCT with CFP or FAF was required to establish the diagnosis of RPE tear, which was accepted when at least two out of three modalities were positive.

### Statistical analysis

The data collected was organized in an Excel spreadsheet. Continuous variables are expressed as mean ± standard deviation. A 2 × 2 table was utilized to analyze the accuracy of the oscillatory sign in identifying RPE tear. Data analysis was conducted using MedCalc software (version 22.03, Ostend, Belgium).

## Results

Electronic medical records from 936 consecutive eyes diagnosed with neovascular age-related macular degeneration (AMD) and assessed using multimodal imaging, including colour fundus photography (CFP), were analyzed. Each of the 936 eyes was systematically evaluated for the presence of the optical coherence tomography (OCT) oscillatory sign, employing the same methodology previously applied to 79 eyes with retinal pigment epithelium (RPE) tears.

Among them, 79 eyes exhibiting RPE tears were identified using OCT and FAF. 38 of 79 eyes with RPE tears showed an oscillatory sign. The mean age of the patients was 75.24 ± 7.94 years (59–98 years). (Table 1) Ten eyes had received prior anti-VEGF injections, with a mean of 1.74 injections prior to a tear. 28 eyes were treatment naïve. Nineteen of the 38 (50%) RPE tears were first detected within the initial two months after symptoms began.

**Table 1:** Showing baseline characteristics of patients with RPE tears showing the Oscillatory sign

Total eyes with RPE tear with oscillatory sign	38
Gender (%)	
Male	23 (60.53%)
Female	15 (39.47%)
Average age ± SD (median) in years	75.24 ± 7.94 (74)
The mean number of injections (median)	1.74 (2)
Mean follow-up in months (median)	12.92 (4) months
Vision at baselines in logMAR (median)	0.73 ± 0.49 (0.6)
Vision at last follow-up in logMAR (median)	0.84 ± 0.42 (0.7)
Number of eyes of RPE tear having an oscillatory sign	38
Cases of RPE tear without the oscillatory sign	41
The average duration of the onset of symptoms before the RPE tear	2.85 ± 2.16 months
RPE tears detected with OCT and FAF	79
RPE tears detected with OCT (Among those who had an oscillatory sign)	38

We evaluated changes in mean BCVA from baseline to the time of RPE tear. Twelve eyes had a decrease in at least one line of vision after developing an RPE tear, and 8 eyes had a decrease in line of vision of more than 2 lines after developing an RPE tear. Most RPE tear cases in our study were associated with spontaneous RPE tears in 23 out of 38 cases (60.52%) compared to 15 cases post-anti-VEGF cases (39.47%). Oscillatory signs demonstrated a high specificity of 98.95% and a sensitivity of 48.10%. The oscillatory sign’s high negative predictive value of 95.39% indicates that it is highly accurate (Tables 2 and 3). Representative cases are shown in Fig. 1.

**Table 2:** A 2 × 2 table was utilized to analyze the diagnostic accuracy of the oscillatory sign in identifying RPE tear

Neovascular AMD cases		Optical Coherence Tomography	
		Oscillatory Sign Positive	Oscillatory Sign Negative
CFP and FAF	RPE tear present	38	41
	No RPE tear	9	848
Total		47	889

**Table 3:** Showing the diagnostic accuracy of the oscillatory sign in identifying RPE tears in nAMD

Statistic	Value	95% Confidence Interval
Sensitivity	48.10%	36.71% to 59.64%
Specificity	98.95%	98.02 to 99.52%
Positive Likelihood Ratio	45.80	22.99 to 91.23
Negative Likelihood Ratio	0.52	0.42 to 0.65
Positive Predictive Value	80.85%	67.95% to 89.37%
Negative Predictive Value	95.39%	94.36% to 96.24%
Accuracy	94.66%	93.02% to 96.01%

## Discussion

This study clearly demonstrates the potential of oscillatory signs in diagnosing RPE tears associated with nAMD. An OCT line or radial scans may not always pass through the latent RPE tear. Once the oscillatory sign is confirmed during OCT capture, we can further refine our focus on the area of the RPE tear. Oscillations are noted on both the steep and slope sides of the tear, as well as on the opposite side of the choroidal neovascular membrane. In our study, we compared RPE scrolling with hyperreflectivity and back shadowing, along with rolled RPE edges, with the concept of oscillation. Similar RPE undulations have been reported in earlier studies [7–9], but the sensitivity and specificity have not been analyzed.

In our study, the oscillatory sign demonstrated low sensitivity (48.10%) but high specificity (98.95%). Therefore, the presence of this sign serves as a highly reliable indicator of an underlying retinal pigment epithelium (RPE) tear. Other studies have reported that RPE undulations are a predictor of RPE tears following anti-VEGF injections; however, we believe the Oscillatory sign may indicate occult RPE tears [10, 11]. Histological studies on RPE tears have confirmed that as scarring begins, RPE undulations can diminish and eventually disappear [12]. The oscillatory sign serves as a valuable indicator for **early detection**, typically manifesting during the acute, pre-scarring phase. The RPE undulations are transient; they diminish and typically resolve within six months. Previously, OCT biomarkers used to predict RPE tears included qualitative factors, such as PED vertical height greater than 450 micrometers: the larger the PED volume, the larger the SRF height [6, 13, 14]. The oscillatory sign may indicate a distinct OCT biomarker, typically observed in the early phase of an RPE tear. The authors hypothesize that the disappearance of oscillatory signs in RPE tear may be due to various factors, including the spontaneous reattachment of the RPE margins, repopulation of the RPE, and scarring [9, 15]. Additionally, the oscillatory sign can be observed in the pre-scarring phase of the RPE tear. In our series, we have noticed that oscillations gradually dampen over 6 months and tend to disappear. With larger RPE tears, the retracted RPE on OCT exhibits an irregularly contoured area; hyperreflectivity and shadowing can decrease over time.

## Strengths and limitations

To our knowledge, this is the largest case series of OCT-based evaluations of RPE tears. This study highlights the high specificity of the Oscillatory sign for RPE tears and its potential as an OCT biomarker. The findings of this study may pave the way for improved diagnosis of RPE tears.

Our study has several limitations that are inherent to retrospective studies. Specifically, our study couldn’t clarify whether the absence of the Oscillatory sign on OCT in some eyes with obvious RPE tears on CFP and FAF is due to remodeling of the RPE tear over time.

## Conclusions

The Oscillatory sign is an OCT biomarker characterized by undulations of the RPE indicative of RPE tears. Although the Oscillatory sign demonstrates high specificity, it exhibits limited sensitivity. The Oscillatory sign possesses a high negative predictive value, effectively excluding acute RPE tears when the sign is not observed. The Oscillatory sign is most valuable for early detection, typically manifesting during the acute, pre-scarring phase. These RPE undulations are transient, generally diminishing and disappearing as the injury progresses to the scarring phase. This biomarker facilitates the diagnosis of occult RPE tears that may not be detected by fundus autofluorescence.

## Data Availability

No datasets were generated or analysed during the current study.
